# Early efforts in modeling the incubation period of infectious diseases with an acute course of illness

**DOI:** 10.1186/1742-7622-4-2

**Published:** 2007-05-11

**Authors:** Hiroshi Nishiura

**Affiliations:** 1 Department of Medical Biometry, University of Tübingen, Westbahnhofstr. 55, Tübingen, D-72070, Germany; 2 Research Center for Tropical Infectious Diseases, Nagasaki University Institute of Tropical Medicine, Sakamoto 1-12-4, Nagasaki, 852-8523, Japan

## Abstract

The incubation period of infectious diseases, the time from infection with a microorganism to onset of disease, is directly relevant to prevention and control. Since explicit models of the incubation period enhance our understanding of the spread of disease, previous classic studies were revisited, focusing on the modeling methods employed and paying particular attention to relatively unknown historical efforts. The earliest study on the incubation period of pandemic influenza was published in 1919, providing estimates of the incubation period of Spanish flu using the daily incidence on ships departing from several ports in Australia. Although the study explicitly dealt with an unknown time of exposure, the assumed periods of exposure, which had an equal probability of infection, were too long, and thus, likely resulted in slight underestimates of the incubation period.

After the suggestion that the incubation period follows lognormal distribution, Japanese epidemiologists extended this assumption to estimates of the time of exposure during a point source outbreak. Although the reason why the incubation period of acute infectious diseases tends to reveal a right-skewed distribution has been explored several times, the validity of the lognormal assumption is yet to be fully clarified. At present, various different distributions are assumed, and the lack of validity in assuming lognormal distribution is particularly apparent in the case of slowly progressing diseases. The present paper indicates that (1) analysis using well-defined short periods of exposure with appropriate statistical methods is critical when the exact time of exposure is unknown, and (2) when assuming a specific distribution for the incubation period, comparisons using different distributions are needed in addition to estimations using different datasets, analyses of the determinants of incubation period, and an understanding of the underlying disease mechanisms.

## Background

The incubation period is defined as the time from exposure to onset of disease [1], and when limited to infectious diseases, corresponds to the time from infection with a microorganism to symptom development. According to a rigorous descriptive review [2], historical descriptions of the incubation period can be traced back to the mid-16th century when Girolamo Fracastoro (Fracastorius) (1478–1553), an Italian physician, documented for the first time the incubation period of rabies in 1546 [3]. The incubation period of infectious diseases ranges from the order of a few hours, which is common for toxic food poisoning, to a few decades as seen in the case of tuberculosis, AIDS and variant Creutzfeldt-Jakob disease (vCJD). Since symptom onset reflects pathogen growth and invasion, excretion of toxins and initiation of host-defense mechanisms, the length of the incubation period varies largely according to the replication rate of the pathogen, the mechanism of disease development, the route of infection and other underlying factors.

During the incubation period of acute infectious diseases, which is subsequently followed by a symptomatic period, it should be noted that the infected host can be infectious. Whereas the incubation and symptomatic periods are distinguished by symptom onset, other epidemiologic terms are distinguished by acquisition of infectiousness. That is, the time from infection to acquisition of infectiousness is referred to as the latent period, which is subsequently followed by the infectious period [4]. These two concepts are clearly separated by definition and are not directly related. The incubation period of infectious diseases offers various insights into clinical and public health practices, as well as being important for epidemiologic and ecological studies.

To enhance our understanding of the incubation period distributions of infectious diseases, it is useful to revisit previous efforts and reassess explicit models. In particular, it is of practical importance to reanalyze historical works to clarify the present day implications. This paper discusses relatively unknown historical efforts, paying particular attention to diseases with an acute course of illness. Previous classic works on models of incubation period are discussed, including the earliest method to estimate the incubation period using incomplete data, the earliest attempt to model the distribution, and estimations of the time of exposure during an outbreak with a common harmful influence and a very brief time of exposure (i.e., a point source outbreak).

## Analysis

### The usefulness of understanding the incubation period

Before entering into details of historical works on the incubation period, the various uses of the incubation period distribution are briefly discussed. Table 1 summarizes a number of common examples, presenting historical as well as recent major uses [1,5-27]; however, it is worth noting that this list does not cover all utilities in full.

**Table 1:** Common uses of the incubation period distribution of infectious diseases

**Major field of use**	**Explanation and example**	**Ref.**
Clinical practice	Rough estimates of the time of exposure of bedside cases (e.g., to determine the causes and/or sources of infection)	5
	Development of a treatment strategy that extends the incubation period (e.g., antiretroviral therapy for HIV/AIDS)	1
	Early projection of disease prognosis when the incubation period is clearly associated with clinical severity (e.g., diseases caused by exotoxin)	6, 7
	Clinical investigations of the impact of infecting dose on the clinical appearance of a disease (i.e., the dose-response mechanism)	8, 9
Public health practice	Determination of the length of quarantine required for a potentially exposed individual (e.g., limiting the movement of those exposed to SARS within a household)	10
Epidemiologic study	Determination of the eradicability of a disease (e.g., determination of the effectiveness of isolation measures)	11
	Estimation of the time of exposure during a point source outbreak (e.g., in identification of the source of infection during large-scale food poisoning)	12
	Determination of the end of a point source outbreak (i.e., statistical tests that determine if case onset is over)	13
	Reconstruction of epidemic curves and short-term predictions of slowly progressing diseases (e.g., backcalculation of HIV/AIDS and prion diseases)	14–23
	Estimation of the transmission potential and infectiousness relative to disease-age (e.g., estimation of the relative infectiousness of smallpox)	24,25
Ecological study	Determination of the adaptation strategy of a parasite (e.g., evolution of vivax malaria owing to seasonal selection pressure)	26,27

In clinical practice, the incubation period is useful not only for making rough guesses as to the causes and sources of infection of individual cases [5], but also for developing treatment strategies to extend the incubation period (e.g., antiretroviral therapy for HIV infection [1]) and for performing early projection of disease prognosis when the incubation period is clearly associated with clinical severity due to dose-response mechanisms (e.g., diseases caused by exotoxin) [6,7]. Moreover, during an outbreak of a newly emerged directly transmitted disease, the incubation period distribution permits determination of the length of quarantine required for a potentially exposed individual (i.e., by restricting movement of an exposed individual for a duration sufficiently longer than the incubation period) [10]. Further, if the time lag between acquiring infectiousness and symptom onset appears long (i.e., if the incubation period is relatively long compared to the latent period), it implies that isolation measures (e.g, restriction of movement until the infectious individual loses infectiousness) are likely to be ineffective, complicating disease control [1,11].

Understanding the incubation period distribution also enables statistical estimation of the time of exposure during a point source outbreak [12] as well as a hypothesis-testing to determine whether the outbreak has ended [13]; the former is discussed below. The distribution is also useful in statistical approaches of epidemic curve reconstruction and short-term predictions of slowly progressing diseases; the backcalculation method uses the incubation period to estimate HIV prevalence and project the future incidence of AIDS [14,15]. During the last decade, this method has also been extended to prion diseases such as Bovine Spongiform Encephalopathy (BSE) [16,17], vCJD [18-22] and Kuru [23]. Although backcalculation is not discussed in this paper, several rigorous reviews have been published with regard to diseases with a long incubation period [15,17,22,28]. This approach has also recently diverged to quantification of the transmission potential of diseases with an acute course of illness [24] and infectiousness relative to disease-age [25]. Moreover, in cases such as the short and long incubation periods of *Plasmodium vivax *malaria in temperate zones, the incubation period also enhances ecological understanding of adaptation strategies; in temperate zones, clearly separate bimodal peaks with approximate lengths of 2 and 50 weeks are observed [26,27], helping malaria transmissions continue over the winter season when transmission is usually greatly reduced due to seasonal entomologic characteristics.

### The earliest model developed using incomplete data

Whereas the incubation period is conveniently extracted from specific data indicating the time of exposure, i.e., experimental inoculation data and case travel histories [2], most infection events are not directly observable for diseases transmitted by non-sexual direct contact. Thus, it is often difficult to determine the incubation period without explicit information of the time of exposure. The majority of epidemiologic data informs us that exposure (i.e., infection) occurred in a defined period, data of which is referred to as interval censored [29]. This is a common concern for acute infectious diseases transmitted by droplets and droplet nuclei and, most noteworthily, was discussed in detail during the outbreak of severe acute respiratory syndrome (SARS) [30,31]. Previous studies on the population dynamics of influenza tend to make assumptions with regard to the incubation period distribution without employing observed data [32,33], perhaps mainly due to difficulties in identifying the time of exposure. The incubation period distribution of influenza remained almost unknown until a recent study reanalyzed the data of influenza transmission on an aircraft with a short duration of flight [34,35]. Assuming Weibull distribution, this study estimated the mean (and standard deviation (SD)) incubation period as 1.48 (0.47) days [35]. Not only was the sample size of the estimate limited (i.e., 37 secondary cases), but since no other estimates are currently available, the present paper revisits a historical study on this topic.

The earliest study concerned with estimating the incubation period of influenza was published by Anderson Gray McKendrick (1876–1943) and J. Morison in the Indian Journal of Medical Sciences in 1919 [36]. Dr. McKendrick, a physician and epidemiologist, applied various mathematical methods to the field of medicine and is a known pioneer in the biomathematics of infectious disease epidemiology [37-39]. Whereas Dr. McKendrick, in collaboration with William Ogilvy Kermack (1898–1970) [40,41], is relatively well known as the first to suggest the deterministic epidemic model given by differential equations, his analysis of the incubation period of Spanish flu preceded this, and remains relatively unknown even among specialists (see Online Additional File 1 for the original). Except for this work, no other historical study on influenza has explicitly accounted for the unknown time of exposure or identified the time of exposure in a specific setting (as in the above mentioned study documenting transmission on an aircraft [34,35]).

**Figure 1:**
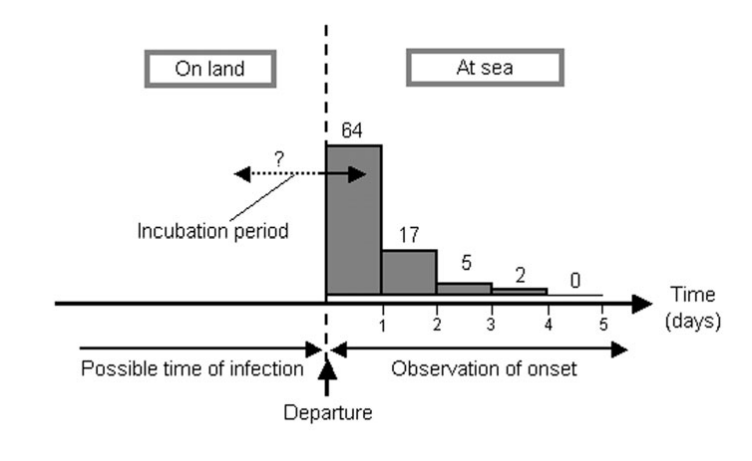
**The relationship between the incubation period and observed onset of influenza after departure from Australia, 1918–19**. Daily frequencies of influenza onset were observed after departure. Those who developed symptoms on board were assumed to have experienced exposure before departure. Since the time of exposure was difficult to identify explicitly, it was necessary to consider all possible times of exposure before departure. Asymptomatic infections and potential secondary transmissions on board were ignored. See supporting material for original descriptions and original data [36, 42, 43].

In Dr. McKendrick's study, an attempt was made to infer the incubation period of pandemic influenza using the daily incidence of cases on ships departing, with incubating individuals, from several ports in Australia. The incidence was recorded according to the date of voyage after departure. The original epidemiologic data was based on observations of 92 departing voyages, summarized by Dr. John Howard Lidgett Cumpston (1880–1954), Director of Quarantine of the Commonwealth of Australia [42] (the original material is available online [43]). In this dataset, onset of 64, 17, 5 and 2 cases was observed on the 1st, 2nd, 3rd and 4th day of voyage, i.e., after departure, on the documented ships (Figure 1). No influenza case developed symptoms on or after the 5th day of voyage and the observed cases were thought to have been exposed to influenza before departure. Since the data for each voyage mainly included only a few initial cases that developed influenza on board, it is assumed that potential secondary transmission on board was negligible, and potential asymptomatic transmission was also ignored (detailed information on the observed and excluded secondary transmissions are documented in the original [43] and Dr. McKendrick also addressed the issue of secondary transmission by limiting the number of cases per ship). Further technical details are given in the Additional File 2.

Using the data in Figure 1 (with a total of *N *cases), the number of cases, *G*(*t*), *t *days after departure was modeled as:

G(t)N=∫−∞0f(t−τ)dτ=1−F(t).
 MathType@MTEF@5@5@+=feaafiart1ev1aaatCvAUfKttLearuWrP9MDH5MBPbIqV92AaeXatLxBI9gBaebbnrfifHhDYfgasaacH8akY=wiFfYdH8Gipec8Eeeu0xXdbba9frFj0=OqFfea0dXdd9vqai=hGuQ8kuc9pgc9s8qqaq=dirpe0xb9q8qiLsFr0=vr0=vr0dc8meaabaqaciaacaGaaeqabaqabeGadaaakeaafaqabeGabaaabaWaaSaaaeaacqWGhbWrcqGGOaakcqWG0baDcqGGPaqkaeaacqWGobGtaaGaeyypa0Zaa8qmaeaacqWGMbGzcqGGOaakcqWG0baDcqGHsisliiGacqWFepaDcqGGPaqkcqWGKbazcqWFepaDaSqaaiabgkHiTiabg6HiLcqaaiabicdaWaqdcqGHRiI8aaGcbaGaeyypa0JaeGymaeJaeyOeI0IaemOrayKaeiikaGIaemiDaqNaeiykaKIaeiOla4caaaaa@4AEE@

where *f*(*t*) and *F*(*t*) are the probability density and cumulative distribution functions of an incubation period of length *t *(see Additional File 2). From this, Dr. McKendrick suggested that the mean incubation period was 32.71 hours, which is consistent with recent estimate [35]. However, this value was likely slightly underestimated, because the model implicitly assumed the possible time of exposure as being from time 0 to infinite before departure; it has been extensively discussed that data assuming long possible periods of exposure is likely to be uninformative. Moreover, in a recent work on SARS concerned with analysis of data with short periods of exposure [44], the equal probability of exposure for each possible date was likely to have overestimated the variance of the incubation period distribution [31]. Thus, to obtain a precise estimate of the incubation period, appropriate censoring methods with well-defined short periods of exposure are needed in addition to a large sample size [30,45]. However, despite these technical concerns, it is remarkable that Dr. McKendrick was able to estimate the incubation period of pandemic influenza considering the unknown time of exposure in the given data.

### Classic right-skewed distribution

**Figure 2:**
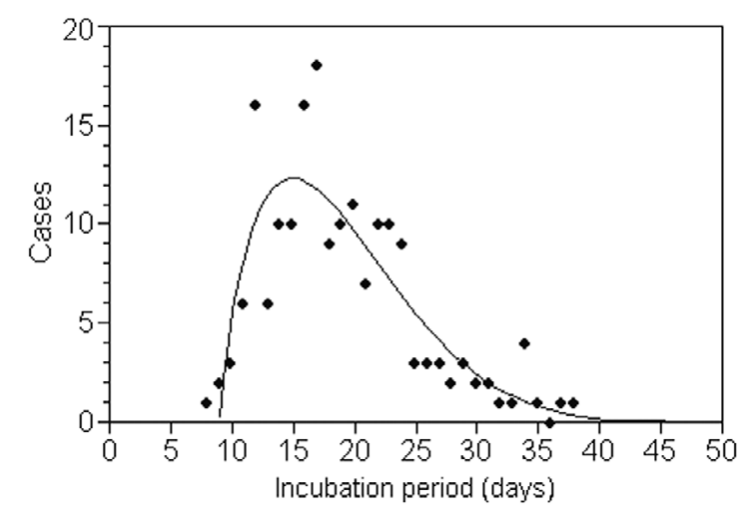
**The incubation period distribution of typhoid fever in Old Salem Chautauqua, 1916, fitted to Pearson's Type I distribution**. The incubation period started at an assumed time of exposure due to a flood that occurred 4 days before closing the water supply to Chautauqua. Since there were 4 possible days of exposure to contaminated water, the original study used the mid-point as a single time point of exposure. See [46] for the original descriptions.

After Dr. McKendrick's initial work and his use of implicit assumptions to determine the incubation period distribution, John R. Miner (1892-unknown), a biologist and epidemiologist at Johns Hopkins University, is believed to have documented the first explicit model of the incubation period distribution [46]. Dr. Miner collected epidemic records of several outbreaks of typhoid fever, claiming that the length of the incubation period clearly differs by source of infection (i.e., comparing water- and food-borne outbreaks, he found that the food-borne outbreaks had a much shorter incubation period, most likely reflecting dose-response phenomena). During his analysis, Dr. Miner paid close attention to variance in the incubation period, describing a distribution that always skewed to the right. In calculating "moments" of the incubation period in a water-borne outbreak at the Old Salem Chautauqua, 1916, he used the following equation to explain the epidemic curve:

y=12.396(1+x5.955)0.8573(1−x40.040)5.7641
 MathType@MTEF@5@5@+=feaafiart1ev1aaatCvAUfKttLearuWrP9MDH5MBPbIqV92AaeXatLxBI9gBaebbnrfifHhDYfgasaacH8akY=wiFfYdH8Gipec8Eeeu0xXdbba9frFj0=OqFfea0dXdd9vqai=hGuQ8kuc9pgc9s8qqaq=dirpe0xb9q8qiLsFr0=vr0=vr0dc8meaabaqaciaacaGaaeqabaqabeGadaaakeaacqWG5bqEcqGH9aqpcqaIXaqmcqaIYaGmcqGGUaGlcqaIZaWmcqaI5aqocqaI2aGndaqadaqaaiabigdaXiabgUcaRmaalaaabaGaemiEaGhabaGaeGynauJaeiOla4IaeGyoaKJaeGynauJaeGynaudaaaGaayjkaiaawMcaamaaCaaaleqabaGaeGimaaJaeiOla4IaeGioaGJaeGynauJaeG4naCJaeG4mamdaaOWaaeWaaeaacqaIXaqmcqGHsisldaWcaaqaaiabdIha4bqaaiabisda0iabicdaWiabc6caUiabicdaWiabisda0iabicdaWaaaaiaawIcacaGLPaaadaahaaWcbeqaaiabiwda1iabc6caUiabiEda3iabiAda2iabisda0iabigdaXaaaaaa@54EE@

where *y *and *x *are the expected number of cases and time after exposure, respectively (Figure 2). The general form of eqn. (2) is referred to as Pearson's type I distribution, which is given by [47]:

y=y0(1+xa1)m1(1−xa2)m2
 MathType@MTEF@5@5@+=feaafiart1ev1aaatCvAUfKttLearuWrP9MDH5MBPbIqV92AaeXatLxBI9gBaebbnrfifHhDYfgasaacH8akY=wiFfYdH8Gipec8Eeeu0xXdbba9frFj0=OqFfea0dXdd9vqai=hGuQ8kuc9pgc9s8qqaq=dirpe0xb9q8qiLsFr0=vr0=vr0dc8meaabaqaciaacaGaaeqabaqabeGadaaakeaacqWG5bqEcqGH9aqpcqWG5bqEdaWgaaWcbaGaeGimaadabeaakmaabmaabaGaeGymaeJaey4kaSYaaSaaaeaacqWG4baEaeaacqWGHbqydaWgaaWcbaGaeGymaedabeaaaaaakiaawIcacaGLPaaadaahaaWcbeqaaiabd2gaTnaaBaaameaacqaIXaqmaeqaaaaakmaabmaabaGaeGymaeJaeyOeI0YaaSaaaeaacqWG4baEaeaacqWGHbqydaWgaaWcbaGaeGOmaidabeaaaaaakiaawIcacaGLPaaadaahaaWcbeqaaiabd2gaTnaaBaaameaacqaIYaGmaeqaaaaaaaa@45E9@

where *m*_1_/*a*_1 _= *m*_2_/*a*_2_. During the early 20th century, it was deemed useful to apply Karl Pearson's (1857–1936) "system of frequency curves" to observed data, because the parameters could be arithmetically obtained from moments determined by the descriptive statistics; a "moment" refers to the expected value of a positive integral power of a random variable (i.e., the *n*th moment of a distribution is the expected value of the *n*th power of the deviation from a fixed value). Among Pearson's curves, type I distribution is the most standard and relatively flexible, and can realize right-skewed distribution [47]. Although no other works concerned with models of the incubation period have been identified, Major Greenwood (1880–1949) applied Pearson's type III distribution to the distribution of the serial interval (i.e., the time from symptom onset in a primary case to symptom onset in a secondary case [48]) of measles within a number of households [49].

### Lognormal distribution proposed by Philip Sartwell

The epidemiologist Philip E. Sartwell (1908–1999), who previously acted as chairman of the Department of Epidemiology, Johns Hopkins School of Hygiene and Public Health, contributed most to the foundation of incubation period distribution modeling [50]. Dr. Sartwell initially found that the incubation period of acute infectious diseases tends to follow lognormal distribution [12], and applied the distribution to various diseases [51,52]. Observing that the distributions often skewed to the right, Dr. Sartwell suggested the use of two parameters (i.e., an estimated "median", which is also the geometric mean due to the characteristics of lognormal distribution, and a "dispersion factor" as a measure of variability) rather than the sample mean and standard deviation. Lognormal distribution has a probability density function (pdf) of:

f(x;μ,σ2)=1xσ2πexp⁡(−(ln⁡(x)−μ)22σ2)
 MathType@MTEF@5@5@+=feaafiart1ev1aaatCvAUfKttLearuWrP9MDH5MBPbIqV92AaeXatLxBI9gBaebbnrfifHhDYfgasaacH8akY=wiFfYdH8Gipec8Eeeu0xXdbba9frFj0=OqFfea0dXdd9vqai=hGuQ8kuc9pgc9s8qqaq=dirpe0xb9q8qiLsFr0=vr0=vr0dc8meaabaqaciaacaGaaeqabaqabeGadaaakeaacqWGMbGzcqGGOaakcqWG4baEcqGG7aWoiiGacqWF8oqBcqGGSaalcqWFdpWCdaahaaWcbeqaaiabikdaYaaakiabcMcaPiabg2da9maalaaabaGaeGymaedabaGaemiEaGNae83Wdm3aaOaaaeaacqaIYaGmcqWFapaCaSqabaaaaOGagiyzauMaeiiEaGNaeiiCaa3aaeWaaeaadaWcaaqaaiabgkHiTiabcIcaOiGbcYgaSjabc6gaUjabcIcaOiabdIha4jabcMcaPiabgkHiTiab=X7aTjabcMcaPmaaCaaaleqabaGaeGOmaidaaaGcbaGaeGOmaiJae83Wdm3aaWbaaSqabeaacqaIYaGmaaaaaaGccaGLOaGaayzkaaaaaa@55B7@

for *x *> 0, where *μ *and *σ *are the mean and standard deviation of the variable's logarithm [53]. The coefficient of variation (CV), a dimensionless number, is a measure of dispersion of the distribution given by:

CV=exp⁡(σ2)−1.
 MathType@MTEF@5@5@+=feaafiart1ev1aaatCvAUfKttLearuWrP9MDH5MBPbIqV92AaeXatLxBI9gBaebbnrfifHhDYfgasaacH8akY=wiFfYdH8Gipec8Eeeu0xXdbba9frFj0=OqFfea0dXdd9vqai=hGuQ8kuc9pgc9s8qqaq=dirpe0xb9q8qiLsFr0=vr0=vr0dc8meaabaqaciaacaGaaeqabaqabeGadaaakeaacqWGdbWqcqWGwbGvcqGH9aqpdaGcaaqaaiGbcwgaLjabcIha4jabcchaWjabcIcaOGGaciab=n8aZnaaCaaaleqabaGaeGOmaidaaOGaeiykaKIaeyOeI0IaeGymaedaleqaaOGaeiOla4caaa@3BB5@

**Figure 3:**
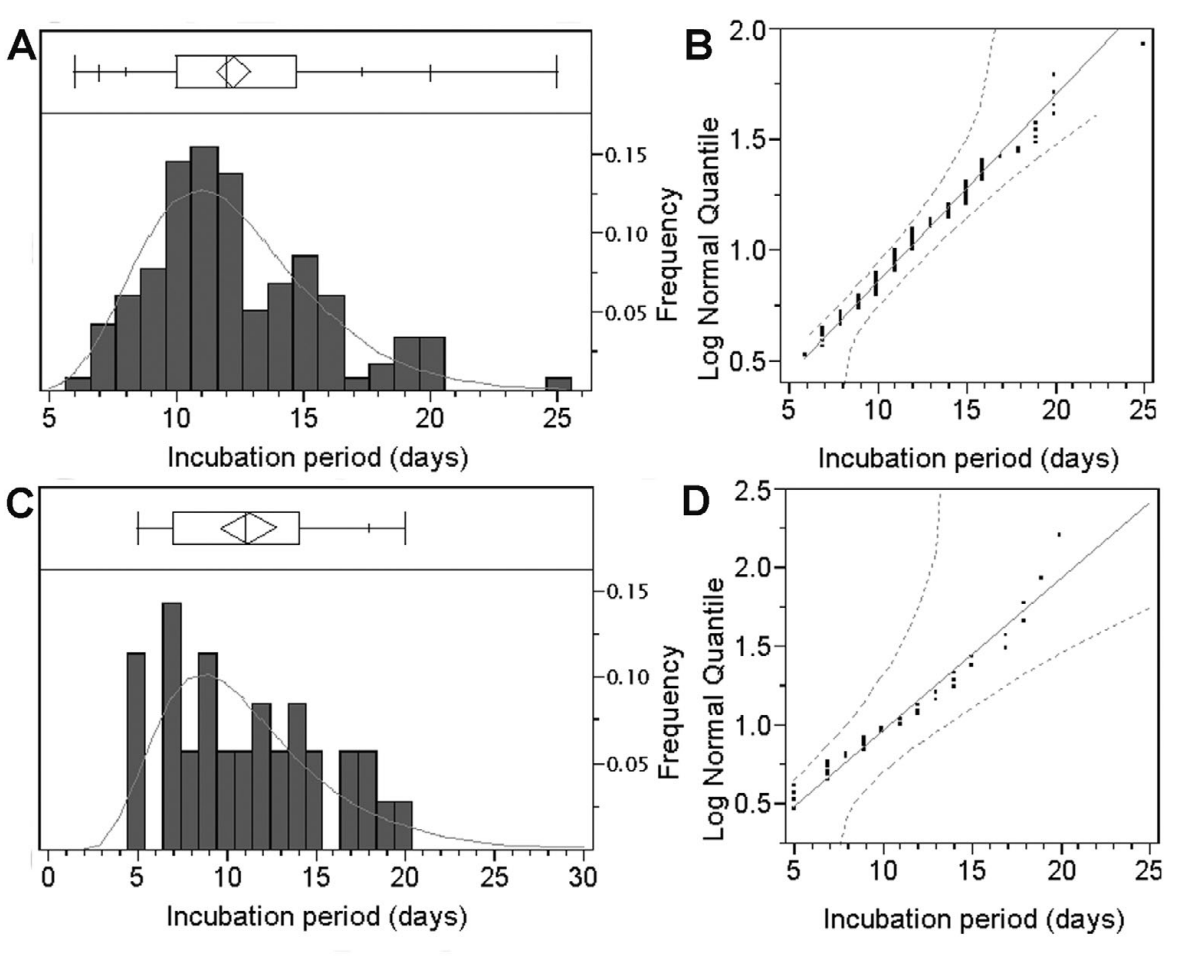
**The incubation period distributions of measles (A and B) and poliomyelitis (C and D) fitted to lognormal distributions**. A &C) Observed frequencies (bars) are compared with predicted frequencies (solid line) based on the maximum likelihood method assuming lognormal distribution. The ends of the box represent the 25th and 75th quantiles (i.e., quartiles), and the line across the middle of the box identifies the median sample value. The means diamond indicates the sample mean and 95% confidence interval. The whiskers extending from both ends show additional quantiles (5th, 10th, 90th and 95th) on the response axis (note: for poliomyelitis (C), some quantiles are overlapping, and therefore only the 90th quantile is displayed). B & D) Lognormal quantile plots of the incubation periods. The diagonal reference lines show the line of fit and the two dashed lines denote confidence limits of 95% equal precision bounds with a = 0.001 and b = 0.99. See [54,55] for original data.

Figure 3 shows the frequency distributions of the incubation periods of measles and poliomyelitis based on careful observations of the times of exposure and onset [54,55] (the maximum likelihood method was used to obtain Fig. 3 and will be discussed later). Both incubation periods were reasonably generalized using lognormal distributions, yielding maximum likelihood estimates of *μ *and CV of 2.47 log(days) and 28.0% and 2.37 log(days) and 47.4%, respectively. The goodness-of-fit to lognormal distribution was then visually assessed by drawing lognormal quantile plots (Figs. 3B and 3D).

Even at present, it is frequently assumed that the incubation period of acute infectious diseases follows lognormal distribution [25,56,57]. Using the lognormal assumption for incubation period, Dr. Sartwell further developed a method to estimate the time of exposure during a point source outbreak [52]. Since the contribution of Dr. Sartwell has been revisited several times elsewhere [2,58] and is relatively well known among experts in this field, similar and directly relevant models proposed by Japanese epidemiologists are discussed in the following.

### Lognormal models proposed by Japanese epidemiologists

**Figure 4:**
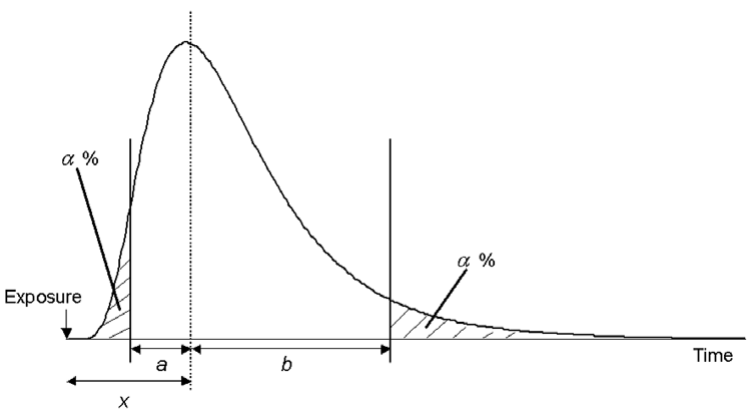
**A method for estimating the time of exposure during a point source outbreak**. The horizontal axis shows the time since exposure and the distribution the frequency of cases according to the time of onset. The vertical dashed line is the median incubation period observed *x *days after exposure. The remaining two vertical lines indicate the times when fractions *α *and 1-*α *of cases developed the disease. The intervals between the median and other two vertical lines represent *a *and *b*, respectively. The illustration was drawn by the author with reference to [59].

Dr. Sartwell's suggestion on the tendency for the incubation period to follow lognormal distribution largely influenced early theoretical epidemiologic studies in Japan, especially those related to estimations of the time of exposure during a point source outbreak. The earliest Japanese work appeared immediately after Dr. Sartwell's first publication and was conducted by Takeshi Hirayama (1923–1995), an epidemiologist who, later in life, worked mainly on the epidemiology of various cancers [59,60]. The theoretical basis of Dr. Hirayama's method is illustrated in Figure 4, the logic of which is explained in the following.

Since all cases in a point source outbreak share the same time of exposure, the epidemic curve, which is drawn according to the time of onset (i.e., incidence), is equivalent to the incubation period distribution (Figure 4). Suppose that the median point of the case frequency was observed *x *days after exposure and, further, that there are *α *percentile points on both sides of the observed distribution (upper and lower percentiles *α*) with the distances from the median to both percentiles points being *a *and *b *days, respectively, the following relationship is given (because the logarithm follows normal distribution):

ln(*x*) - ln(*x *- *a*) = ln(*x *+ *b*) - ln(*x*).

This is rearranged as:

xx−a=x+bx
 MathType@MTEF@5@5@+=feaafiart1ev1aaatCvAUfKttLearuWrP9MDH5MBPbIqV92AaeXatLxBI9gBaebbnrfifHhDYfgasaacH8akY=wiFfYdH8Gipec8Eeeu0xXdbba9frFj0=OqFfea0dXdd9vqai=hGuQ8kuc9pgc9s8qqaq=dirpe0xb9q8qiLsFr0=vr0=vr0dc8meaabaqaciaacaGaaeqabaqabeGadaaakeaadaWcaaqaaiabdIha4bqaaiabdIha4jabgkHiTiabdggaHbaacqGH9aqpdaWcaaqaaiabdIha4jabgUcaRiabdkgaIbqaaiabdIha4baaaaa@381D@

Consequently, the time of exposure can be inferred using the distance from the time of exposure to the median, *x*, by taking the distances to any equal percentiles on both sides:

x^=abb−a
 MathType@MTEF@5@5@+=feaafiart1ev1aaatCvAUfKttLearuWrP9MDH5MBPbIqV92AaeXatLxBI9gBaebbnrfifHhDYfgasaacH8akY=wiFfYdH8Gipec8Eeeu0xXdbba9frFj0=OqFfea0dXdd9vqai=hGuQ8kuc9pgc9s8qqaq=dirpe0xb9q8qiLsFr0=vr0=vr0dc8meaabaqaciaacaGaaeqabaqabeGadaaakeaacuWG4baEgaqcaiabg2da9maalaaabaGaemyyaeMaemOyaigabaGaemOyaiMaeyOeI0Iaemyyaegaaaaa@3568@

This estimator is theoretically the same as that suggested by Dr. Sartwell in his later work [52]. Although this model can theoretically assume any *α *(for 0 <*α *< 0.5), Dr. Hirayama implicitly suggested the use of *α *= 0.16 to obtain a precise estimate of *x *and small SD, but this suggestion was made based on observational experience alone and analytical expression for the SD was unfortunately lacking. Since recall bias is unavoidable in retrospective epidemiologic studies of food poisoning requiring huge efforts of food traceback [61], this method appears to be very useful in determining the most plausible time of exposure and narrowing down the amount of information to be traced. A similar method has been applied to the epidemiology of cancer and other chronic diseases [62,63].

Another lognormal assumption was made by a research group on Theoretical Epidemiology at Osaka City University Medical School, mainly and initially led by Kazuya Horiuchi and Hiroshi Sugiyama [64,65]. The methodology has been frequently applied to field data in Japan [66,67] and is relatively well known among Japanese public health workers [68-70]. Dr. Horiuchi examined the validity and precision of Dr. Hirayama's method using Monte Carlo simulations, claiming that the method could be improved further [71] and suggesting that the incubation period should be assumed to follow "non-central" lognormal distribution when an epidemic curve is used [64]. That is, although Dr. Hirayama used the distance from the time of exposure to the median (*x *in eqn. (6)), this is unknown information in field observations, and thus, Dr. Horiuchi and his colleagues suggested the use of *x*-*C*, where *C *is the time of exposure. This permitted the more convenient use of calendar time. For example, let *X *be a random variable following non-central lognormal distribution, ln(*X*-*C*) should follow a normal distribution, N(*μ*, *σ *^2^), and consequently, the following *t *becomes a linear function of ln(*X*-*C*):

t=ln⁡(X−C)−μσ
 MathType@MTEF@5@5@+=feaafiart1ev1aaatCvAUfKttLearuWrP9MDH5MBPbIqV92AaeXatLxBI9gBaebbnrfifHhDYfgasaacH8akY=wiFfYdH8Gipec8Eeeu0xXdbba9frFj0=OqFfea0dXdd9vqai=hGuQ8kuc9pgc9s8qqaq=dirpe0xb9q8qiLsFr0=vr0=vr0dc8meaabaqaciaacaGaaeqabaqabeGadaaakeaacqWG0baDcqGH9aqpdaWcaaqaaiGbcYgaSjabc6gaUjabcIcaOiabdIfayjabgkHiTiabdoeadjabcMcaPiabgkHiTGGaciab=X7aTbqaaiab=n8aZbaaaaa@3B48@

When we assume that the random variable *X *is a function of *t*, eqn.(9) can be rearranged as:

*X*(*t*) = exp(*σt *+ *μ*) + *C*.

Further, considering different values of *t*, e.g., *t*+*h*, yields:

X(t+h)=exp⁡{σ(t+h)+μ}+C=exp⁡(σh)X(t)+C(1−exp⁡(σh)).
 MathType@MTEF@5@5@+=feaafiart1ev1aaatCvAUfKttLearuWrP9MDH5MBPbIqV92AaeXatLxBI9gBaebbnrfifHhDYfgasaacH8akY=wiFfYdH8Gipec8Eeeu0xXdbba9frFj0=OqFfea0dXdd9vqai=hGuQ8kuc9pgc9s8qqaq=dirpe0xb9q8qiLsFr0=vr0=vr0dc8meaabaqaciaacaGaaeqabaqabeGadaaakeaafaqadeGabaaabaGaemiwaGLaeiikaGIaemiDaqNaey4kaSIaemiAaGMaeiykaKIaeyypa0JagiyzauMaeiiEaGNaeiiCaa3aaiWaaeaaiiGacqWFdpWCcqGGOaakcqWG0baDcqGHRaWkcqWGObaAcqGGPaqkcqGHRaWkcqWF8oqBaiaawUhacaGL9baacqGHRaWkcqWGdbWqaeaacqGH9aqpcyGGLbqzcqGG4baEcqGGWbaCcqGGOaakcqWFdpWCcqWGObaAcqGGPaqkcqWGybawcqGGOaakcqWG0baDcqGGPaqkcqGHRaWkcqWGdbWqcqGGOaakcqaIXaqmcqGHsislcyGGLbqzcqGG4baEcqGGWbaCcqGGOaakcqWFdpWCcqWGObaAcqGGPaqkcqGGPaqkcqGGUaGlaaaaaa@6429@

Using eqns.(10) and (11), an estimate of *C *was obtained by graphically plotting these two functions on vertical and horizontal axes, respectively, and then finding the intersect. Estimation of the time of exposure using similar assumptions was extensively discussed in Japan during the 1950s and 60s. These discussions included the following: (i) the definition of the incubation period (e.g., which to use as the time of onset during a food-borne outbreak, the onset of diarrhea or fever? [72,73]), (ii) extension of the estimation method when the data is truncated [68], (iii) the influence of host- and pathogen-related factors and routes of infection on the incubation period [74], and (iv) outbreaks that include cases resulting from human-to-human secondary transmissions (e.g., shigellosis [75]).

### More modern studies employing lognormal distribution

Although the studies described above have offered useful and practical methods based on an understanding of the characteristics of lognormal distribution, the classic methods likely included sampling errors and did not achieve acceptable precision. Indeed, it has been pointed out that the estimates obtained using the methods of Drs. Sartwell and Hirayama largely depend on optional percentile points, *α *[76], while that proposed by Dr. Horiuchi and his colleagues is also thought to be highly sensitive to an optional value, *h *[77]. Thus, estimates of the time of exposure should be addressed statistically by precise solution of the three-parameter lognormal distribution [78,79]. Accordingly, in line with this, the maximum likelihood method was proposed [77,80,81]. Although Dr. Hill was the first to document the application of the maximum likelihood method [80], it unfortunately remained relatively unknown, especially among Japanese epidemiologists, until Toshiro Tango, a statistician at the National Institute of Public Health, Japan, attempted to propagate the method and propose reasonable estimators during the 1990s [77,81]. Let *γ *be the time of exposure, the pdf of the three-parameter lognormal distribution is given by:

f(x;γ,μ,σ2)=1(x−γ)σ2πexp⁡(−(ln⁡(x−γ)−μ)22σ2)
 MathType@MTEF@5@5@+=feaafiart1ev1aaatCvAUfKttLearuWrP9MDH5MBPbIqV92AaeXatLxBI9gBaebbnrfifHhDYfgasaacH8akY=wiFfYdH8Gipec8Eeeu0xXdbba9frFj0=OqFfea0dXdd9vqai=hGuQ8kuc9pgc9s8qqaq=dirpe0xb9q8qiLsFr0=vr0=vr0dc8meaabaqaciaacaGaaeqabaqabeGadaaakeaacqWGMbGzcqGGOaakcqWG4baEcqGG7aWoiiGacqWFZoWzcqGGSaalcqWF8oqBcqGGSaalcqWFdpWCdaahaaWcbeqaaiabikdaYaaakiabcMcaPiabg2da9maalaaabaGaeGymaedabaGaeiikaGIaemiEaGNaeyOeI0Iae83SdCMaeiykaKIae83Wdm3aaOaaaeaacqaIYaGmcqWFapaCaSqabaaaaOGagiyzauMaeiiEaGNaeiiCaa3aaeWaaeaadaWcaaqaaiabgkHiTiabcIcaOiGbcYgaSjabc6gaUjabcIcaOiabdIha4jabgkHiTiab=n7aNjabcMcaPiabgkHiTiab=X7aTjabcMcaPmaaCaaaleqabaGaeGOmaidaaaGcbaGaeGOmaiJae83Wdm3aaWbaaSqabeaacqaIYaGmaaaaaaGccaGLOaGaayzkaaaaaa@5F09@

for *x *> *γ *. Other parameters are as in eqn.(4). The likelihood function is given by the pdf:

L(γ,μ,σ2)=∏i=1nf(xi;γ,μ,σ2)
 MathType@MTEF@5@5@+=feaafiart1ev1aaatCvAUfKttLearuWrP9MDH5MBPbIqV92AaeXatLxBI9gBaebbnrfifHhDYfgasaacH8akY=wiFfYdH8Gipec8Eeeu0xXdbba9frFj0=OqFfea0dXdd9vqai=hGuQ8kuc9pgc9s8qqaq=dirpe0xb9q8qiLsFr0=vr0=vr0dc8meaabaqaciaacaGaaeqabaqabeGadaaakeaacqWGmbatcqGGOaakiiGacqWFZoWzcqGGSaalcqWF8oqBcqGGSaalcqWFdpWCdaahaaWcbeqaaiabikdaYaaakiabcMcaPiabg2da9maarahabaGaemOzayMaeiikaGIaemiEaG3aaSbaaSqaaiabdMgaPbqabaGccqGG7aWocqWFZoWzcqGGSaalcqWF8oqBcqGGSaalcqWFdpWCdaahaaWcbeqaaiabikdaYaaakiabcMcaPaWcbaGaemyAaKMaeyypa0JaeGymaedabaGaemOBa4ganiabg+Givdaaaa@4E7B@

where *n *is the total number of cases observed in an outbreak. Although maximum likelihood estimates of *γ*, *μ *and *σ *are obtained by minimizing the negative logarithm of eqn.(13), it is often the case that the iteration does not converge [82], and thus, Dr. Tango proposed his estimators [77,81]. Assuming that *γ *is known, maximum likelihood estimators of *μ *and *σ *are given by:

μ^(γ)=1n∑1nln⁡(xi−γ),
 MathType@MTEF@5@5@+=feaafiart1ev1aaatCvAUfKttLearuWrP9MDH5MBPbIqV92AaeXatLxBI9gBaebbnrfifHhDYfgasaacH8akY=wiFfYdH8Gipec8Eeeu0xXdbba9frFj0=OqFfea0dXdd9vqai=hGuQ8kuc9pgc9s8qqaq=dirpe0xb9q8qiLsFr0=vr0=vr0dc8meaabaqaciaacaGaaeqabaqabeGadaaakeaaiiGacuWF8oqBgaqcaiabcIcaOiab=n7aNjabcMcaPiabg2da9maalaaabaGaeGymaedabaGaemOBa4gaamaaqahabaGagiiBaWMaeiOBa4MaeiikaGIaemiEaG3aaSbaaSqaaiabdMgaPbqabaGccqGHsislcqWFZoWzcqGGPaqkaSqaaiabigdaXaqaaiabd6gaUbqdcqGHris5aOGaeiilaWcaaa@44CA@

σ^2(γ)=1n∑1n{ln⁡(xi−γ)−μ^(γ)}2.
 MathType@MTEF@5@5@+=feaafiart1ev1aaatCvAUfKttLearuWrP9MDH5MBPbIqV92AaeXatLxBI9gBaebbnrfifHhDYfgasaacH8akY=wiFfYdH8Gipec8Eeeu0xXdbba9frFj0=OqFfea0dXdd9vqai=hGuQ8kuc9pgc9s8qqaq=dirpe0xb9q8qiLsFr0=vr0=vr0dc8meaabaqaciaacaGaaeqabaqabeGadaaakeaaiiGacuWFdpWCgaqcamaaCaaaleqabaGaeGOmaidaaOGaeiikaGIae83SdCMaeiykaKIaeyypa0ZaaSaaaeaacqaIXaqmaeaacqWGUbGBaaWaaabCaeaadaGadaqaaiGbcYgaSjabc6gaUjabcIcaOiabdIha4naaBaaaleaacqWGPbqAaeqaaOGaeyOeI0Iae83SdCMaeiykaKIaeyOeI0Iaf8hVd0MbaKaacqGGOaakcqWFZoWzcqGGPaqkaiaawUhacaGL9baadaahaaWcbeqaaiabikdaYaaaaeaacqaIXaqmaeaacqWGUbGBa0GaeyyeIuoakiabc6caUaaa@4F4B@

Using these, the maximum log likelihood is given as a function of *γ *:

ln⁡L∗∗(γ)≡ln⁡sup⁡μ,σ2L(γ,μ,σ2)=−n(μ^(γ)+ln⁡σ^(γ))−n2(1+log⁡(2π))
 MathType@MTEF@5@5@+=feaafiart1ev1aaatCvAUfKttLearuWrP9MDH5MBPbIqV92AaeXatLxBI9gBaebbnrfifHhDYfgasaacH8akY=wiFfYdH8Gipec8Eeeu0xXdbba9frFj0=OqFfea0dXdd9vqai=hGuQ8kuc9pgc9s8qqaq=dirpe0xb9q8qiLsFr0=vr0=vr0dc8meaabaqaciaacaGaaeqabaqabeGadaaakeaacyGGSbaBcqGGUbGBcqWGmbatdaahaaWcbeqaaiabgEHiQiabgEHiQaaakiabcIcaOGGaciab=n7aNjabcMcaPiabggMi6kGbcYgaSjabc6gaUnaaxababaGagi4CamNaeiyDauNaeiiCaahaleaacqWF8oqBcqGGSaalcqWFdpWCdaahaaadbeqaaiabikdaYaaaaSqabaGccqWGmbatcqGGOaakcqWFZoWzcqGGSaalcqWF8oqBcqGGSaalcqWFdpWCdaahaaWcbeqaaiabikdaYaaakiabcMcaPiabg2da9iabgkHiTiabd6gaUjabcIcaOiqb=X7aTzaajaGaeiikaGIae83SdCMaeiykaKIaey4kaSIagiiBaWMaeiOBa4Maf83WdmNbaKaacqGGOaakcqWFZoWzcqGGPaqkcqGGPaqkcqGHsisldaWcaaqaaiabd6gaUbqaaiabikdaYaaacqGGOaakcqaIXaqmcqGHRaWkcyGGSbaBcqGGVbWBcqGGNbWzcqGGOaakcqaIYaGmcqWFapaCcqGGPaqkcqGGPaqkaaa@71A6@

which is the profile likelihood of *γ *; the estimate of *γ *maximizes eqn.(16). A Bayesian method was also proposed by Dr. Hill, in addition to the maximum likelihood method [80].

### The validity of a lognormal assumption

Despite rigorous studies, it should be noted that we have limited explicit explanations for the biological validity of assuming lognormal distribution for the incubation period. The fundamental biological reason to assume lognormal distribution is related to an inoculation study of ectromelia virus (mouse pox) [83], which suggested exponential growth of pathogens within the host during the initial phase. Another similar study suggested that a fixed threshold likely exists when the host response is observed [84]. Based on these findings, pathogen growth in inoculation experiments was modeled using the birth-death process, supporting right skewed distribution of the incubation period and its long tail [85-87]. Also, given similar results from further birth-death process models [76,88] and another previous model [89], what we have learnt to date can be described as follows: if the growth rate of a microorganism is implicitly assumed to follow normal distribution, and if there is a fixed threshold of pathogen load at which symptoms are revealed due to the host response, exponential growth of microorganisms should result in an incubation period sufficiently approximated by lognormal distribution.

Given the above reasonable explanations, a previous Japanese study examined 86 outbreak records for which the date of infection was known and the population exposed was homogeneous [90]. By assessing the goodness-of-fit, 61 out of the 86 examples (70.9%) were accepted as lognormal at a 5% level of significance or better, from which it was concluded that, in general, lognormal distribution represents the incubation period of acute infectious diseases [90,91]. Through such efforts, the validity of the lognormal assumption has been supported by the accumulated experience of Dr. Sartwell and the above mentioned Japanese epidemiologists. It may also be true that the lognormal distribution was preferred because of its statistical usefulness (as described in the above Japanese study). However, the host-defense mechanism, which is almost entirely responsible for symptom onset, was later shown to be far more complex than previously expected. For example, fever is induced by very complex reactions and by several factors including circulating cytokines such as interluekin-2 [92]. Thus, whereas lognormal distribution may be applied to the incubation periods of many acute infectious diseases, it is necessary to bear in mind that the assumption is supported only by previous experience.

### A further critique of the lognormal assumption

**Figure 5:**
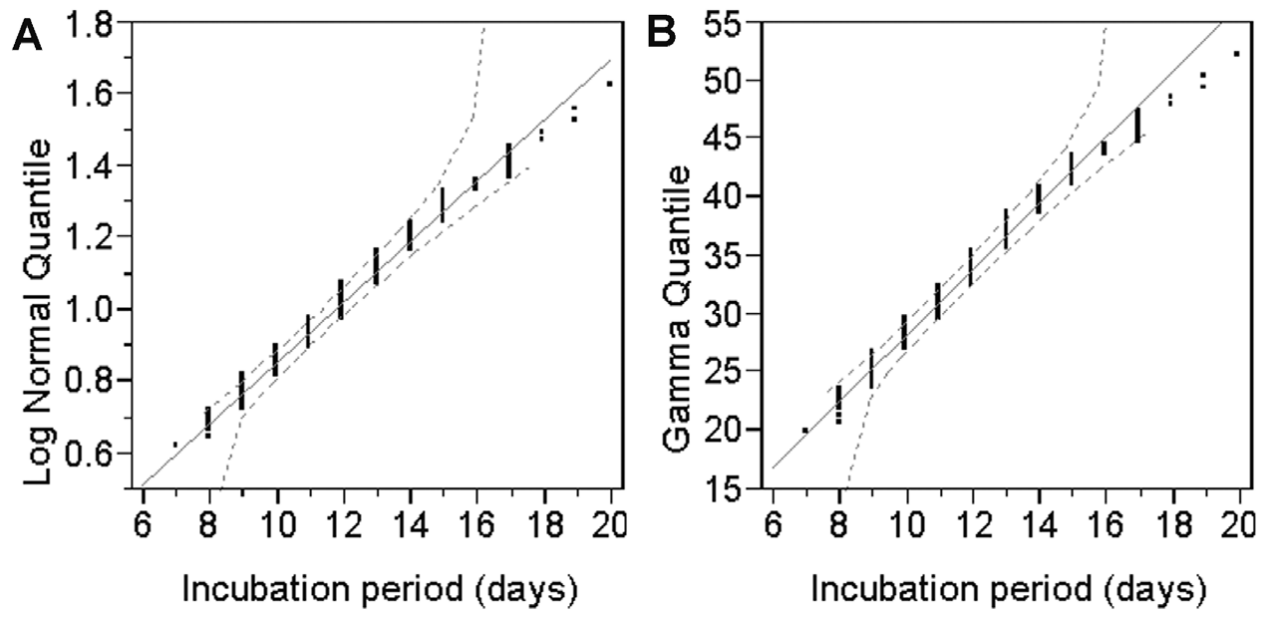
**Comparison of the quantile plots for the incubation period distributions of smallpox assuming (A) lognormal and (B) gamma distribution**. The diagonal reference lines show the line of fit. The maximum likelihood estimates of the mean (*μ*) and standard deviation (*σ*) for lognormal distribution were 2.47 (95% CI: 29.1, 38.6) and 0.36 (0.31, 0.41), respectively. The shape (*α*) and scale (*β*) parameters for gamma distribution were estimated as 33.6 (95% CI: 29.1, 38.6) and 0.36 (0.31, 0.41), respectively. See [96] for detailed descriptions.

Until recently, the validity of assuming lognormal distribution has not been explicitly compared with that of other distributions. As discussed above, Weibull distribution with a threshold (i.e., three parameter Weibull distribution) was assumed for the incubation period of influenza [35]. Such study indicates that a simple lognormal assumption does not always hold in practice. Other studies have assumed gamma distribution for the incubation periods of SARS and smallpox [24,30,93-95], and regarding the latter, lognormal distribution has also been assumed [25,96]. Figure 5 compares the quantile plots of lognormal and gamma distribution for the incubation period of smallpox, showing that both fit almost equally well with the observed data. For both distributions, the χ^2 ^goodness-of-fit test revealed no significant deviation between the observed and predicted values (χ^2^_12 _= 11.6, p = 0.48 and χ^2^_12 _= 16.8, p = 0.16 for lognormal and gamma distributions, respectively). However, two-parameter Weibull distribution did not represent well the probability density functions of the incubation period (p < 0.001). These discussions imply that comparisons using different distributions are needed; it is important to at least compare the goodness-of-fit of different and arbitrarily chosen distributions for acute infectious diseases.

The validity of lognormal assumption is particularly lacking for slowly progressing diseases. One important reason for this is that the mechanisms of disease development for AIDS and prion diseases, for example, are far more complicated than those of acute infectious diseases. In the case of AIDS, where Weibull distribution is frequently assumed for the incubation period [15], symptom onset is induced by immunodeficiency resulting from HIV infection followed by various opportunistic infections. For BSE and vCJD, various distributions have been assumed for the backcalculation, permitting some uncertainty analyses [19,20,22,97]. Although the disease mechanisms of vCJD are yet to be clarified, considering within-host dynamics it is evident that the incubation period cannot be explained by the above simple explanation [98,99]. That is, for these diseases, the above mentioned explanation for the lognormal assumption is not justified, and thus, the choice of distribution for the incubation period needs to be carefully assessed using sensitivity and uncertainty analyses. Indeed, various right skewed distributions are often used in sensitivity analysis, revealing whether or not the final results depend on the arbitrarily chosen standard distribution for the incubation period [19,20,97].

Two conclusions can be drawn from the above discussions. First, the lognormal assumption does not always hold. Thus, as far as we continue to rely on observed frequencies and arbitrarily chosen specific distributions, it is essential that comparisons using different distributions are made; any assumptions should be explicitly evaluated by means of significance tests and visual assessments. Second, the biological validity of assuming specific distributions for the incubation period remains an open question [100,101], and thus, further information is needed. For example, within-host dynamics would help clarify disease onset mechanisms in the most explicit way [102]. Moreover, if information associated with within-host dynamics is not available, an accumulation of distributions obtained using different datasets would be of interest, as would examination of various characteristic factors (e.g., dose-response mechanisms [6,7,9], and variable susceptibility due to age, race and genetic factors (for example, see [45,94])).

## Conclusion

The present study revisited previous works concerned with models of the incubation period of acute infectious diseases. In particular, the following were highlighted: (i) the earliest modeling effort conducted using incomplete data of a pandemic influenza, (ii) the explicit distribution of the incubation period, (iii) the application of a lognormal assumption to estimations of the time of exposure during a point source outbreak, and (iv) the validity of assuming lognormal distribution for the incubation period. Although it was not highlighted in the present paper, Norman T. J. Bailey also formed a framework using a chain binomial model, which is useful for household transmission data [103,104]. This method estimates the incubation period as the sum of the mean latent period, which follows normal distribution, and a further fixed infectious period; however, the estimated period does not precisely imply the incubation period, but rather is closer to what is presently referred to as the serial interval [48,105]. That is, the incubation period that can be extracted from household transmission data remains to be clarified.

The lessons that can be learnt from the presented discussion are as follows: (I) although it is historically remarkable that the incubation period of pandemic influenza was assessed based on an explicit understanding of an unknown time of exposure, the assumed periods of exposure were too long and equal probability of exposure was assumed for each possible date. Well-defined short periods of exposure are needed to decipher the incubation period distribution using appropriate statistical methods. Taking this point into account will be critically important in estimating the incubation period of newly emerging diseases in the future. (II) The epidemiologic usefulness of the lognormal assumption was highlighted with respect to the basic characteristics of lognormal distribution, but this assumption is likely to remain unwarrantable until details of disease mechanisms are fully clarified; thus, this assumption may be merely an approximation of the right-skewed distribution. For example, considering the mechanisms of disease development, the lognormal assumption does not hold for HIV/AIDS and prion diseases. However, this limitation of the lognormal assumption does not imply that such approximation of the incubation period distribution is meaningless. Rather, it suggests that when parametric models are assumed, it is at least necessary to compare the goodness-of-fit for several distributions in order to overcome some of the uncertainty. Various datasets on the same disease would also help assess the uncertainty. Further, it would be informative if the determinants could be clarified even by simple stratifications (e.g., with respect to sex, age and genetic factors). Ideally, assumptions in the future should be supported by a detailed understanding of the underlying disease mechanisms provided by observations of within-host dynamics. Since the incubation period of infectious diseases is directly relevant to prevention and control, and because such knowledge can enhance our theoretical understanding of the spread of disease, further clarifications of the above points are deemed necessary.

## Abbreviations

AIDS – Acquired Immunodeficiency Syndrome

CV – Coefficient of variation

HIV – Human Immunodeficiency Virus

pdf – Probability density function

SARS – Severe Acute Respiratory Syndrome

vCJD – variant Creutzfeldt-Jakob disease

## Competing interests

The author(s) declare that they have no competing interests.

## Authors' contributions

HN carried out paper reviews, proposed the study, performed mathematical analyses and drafted the manuscript. The author has read and approved the final manuscript.

## Supplementary Material

Additional File 1 Original ref. [36] is provided in pdf format. Click here for file

Additional File 2 Technical details of the estimated incubation period of pandemic influenza are provided in pdf format. Click here for file
