# Supplementary material for: Early efforts in modeling the incubation period of infectious diseases with an acute course of illness
Source: Emerg Themes Epidemiol. 2007 May 11;4:2. doi: 10.1186/1742-7622-4-2 (PMC1884151; doi:10.1186/1742-7622-4-2)
Supplement: Additional File 1 — Original ref. [36] is provided in pdf format. [file 1742-7622-4-2-S1.pdf]

THE DETERMINATION OF INCUBATION PERIODS FROM MARITIME STATISTICS, WITH PARTICULAR REFERENCE TO THE INCUBATION PERIOD OF INFLUENZA.

BY

MAJOR A. G. MCKENDRICK, M.B., F.R.S.E., I.M.S.,

Director, Pasteur Institute of India, Kasauli,

AND

MAJOR J. MORISON, M.B., D.P.H., I.M.S.,

On special duty, Influenza Enquiry.

[Received for publication, October 24, 1919.]

THE numerous reports which have been issued concerning the recent influenza pandemic, compiled with great care, and emanating from all parts of the world, provide a rich mine of information regarding the epidemiological features of the disease. In one of these ('Influenza and Maritime Quarantine in Australia,' Service pamphlet No. 18), Dr. F. H. Clumpston, Director of Quarantine of the Commonwealth of Australia, deals with the period of quarantine which should be enforced in order to prevent the introduction of the disease into a non-infected country. The report records many observations on the incidence, spread, and decline of epidemics on board ships. The figures

are of peculiar interest in that every member of these ship populations was under observation throughout the course of the epidemic.

The particular statistics with which we propose to deal in the present communication refer to the number of days after leaving port on which the first case of influenza appeared. We shall show how it is possible from statistics of this nature to deduce the mean incubation period of a disease, and also the expected frequency of occurrence of all incubation periods. That is to say, we shall calculate the relative numbers of cases which would be expected to have incubation periods of 1, 2, 3......days respectively.

The method is, of course, general, and can be applied to similar figures relating to any disease. We shall apply it, in the present instance, to the figures for influenza collected by Dr. Clumpston, but it must be clearly realised that too much stress should not be laid on numerical values obtained from a single set of observations.

The material with which we have to deal relates to 'all the interstate or coastal vessels which became infected between 1st January and 30th April, 1919.' These coastal vessels were constantly inspected from port to port, and were therefore under conditions 'such as to permit of more accurate observation' regarding the occurrence of even single cases than was possible in the case of overseas vessels. The ships varied in size from tug boats to liners of over 10,000 tons, and their crews and passengers ranged in number from 6 to 1,189 individuals. Of the total vessels examined, 92 were found to have been infected. 'There were 64 in which the incubation period appeared to be within one day after leaving port or during their stay in port, there were 17 in which the incubation period appeared to be two days, 5 in which it appeared to be three days, and 2 in which it appeared to be four days.' The first day is the day after the vessel leaves port, but it may also include a part of the day in which the vessel left port; thus in an extreme case, the so-called first day might refer to a period of 36 hours. We shall however in making numerical computations take the figures as they stand, that is to say, 64 on the first day, 17 on the second, 5 on the third, and 2 on the fourth day, but it must be fully realised that the accuracy of the numerical results which we shall obtain depends upon the validity of this simplification.

We shall also, in the first instance, assume that in each ship, only a single case of influenza was taken on board; and then later, by statistical grouping, we shall examine the validity of this assumption.

The problem presents certain points of interest:—

1. If the first case developed symptoms on the first day after leaving port, it need not necessarily have had a period of incubation of one day. The patient might have been infected 0, 1, 2,......days before leaving port. Similarly, if the first case developed symptoms on the second day out, the only incubation period which is excluded is that of one day—and so on.

2. That a case had an incubation period of, say, three days implies that he did not show symptoms on the first and the second days, or, in other words, the probability of an incubation period of three days is the probability that symptoms appeared *for the first time* on the third day.

3. We are thus thrown back to the consideration of a fundamental probability; which is the probability that a case showed symptoms on any particular day, irrespective of whether he did or did not show symptoms on the previous days.

4. In what follows the term 'case of influenza' is used to denote a person who ultimately developed symptoms. Persons, who, although infected, escaped the disease, are excluded.

Let p_r denote the fundamental probability to which we have referred. That is to say, let $p_1, p_2, p_3, \ldots\ldots p_r$ denote the probabilities that a case showed symptoms on the 1st, 2nd, 3rd......*r*th days respectively.

Then $(1-p_1)$, $(1-p_2)$, $(1-p_3)\ldots(1-p_r)$ are the probabilities that he did not show symptoms on the respective days.

That a case showed symptoms *for the first time* on, say, the third day—or, in other words, that he had an incubation period of 3 days—implies that symptoms failed to appear on the first and second days, but appeared on the third. The probability of such an occurrence is then $(1-p_1)\ (1-p_2)\ p_3$.

Thus if Z_1, $Z_2, Z_3 \ldots Z_r$ denote the probabilities of occurrence of incubation periods of 1, 2, 3,........*r* days respectively.

$$\left.\begin{aligned} Z_1 &= p_1, \\ Z_2 &= (1-p_1)\ p_2, \\ Z_3 &= (1-p_1)\ (1-p_2)\ p_3, \\ \text{and in general}\quad Z_r &= (1-p_1)\ (1-p_2)\ldots(1-p_{r-1})\ p_r. \end{aligned}\right\}(1)$$

If the first case after leaving port developed symptoms on the 1st day, he may have had an incubation period of 1 or 2 or 3 or....*r* days;

on the 2nd day, he may have had an incubation period of 2 or 3 or ....r days;

on the 3rd day, he may have had an incubation period of 3 or 4 or....r days.

In other words, a first case with a

1	day incubation period	can occur	only	on the	1st day,
2	days ,,	,,	,,	,,	1st or 2nd days,
3	,, ,,	,,	,,	,,	1st or 2nd or 3rd days, etc.

Thus the total number of *ways* in which a first case can occur is

1 way,	with an incubation	period	of	1 day,
2 ways,	,, ,,	,,	,,	2 days,
3 ,,	,, ,,	,,	,,	3 days, etc.

Now the probability of a first case occurring on any particular day is the sum of the probabilities of the ways in which it can occur on that particular day, divided by the sum of the probabilities of the ways in which it can occur on all the days.

Hence the probability of occurrence of a first case

on the 1st day is $\dfrac{Z_1 + Z_2 + Z_3 + \dots}{1Z_1 + 2Z_2 + 3Z_3 + \dots}$,

on the 2nd day is $\dfrac{Z_2 + Z_3 + \dots}{1Z_1 + 2Z_2 + 3Z_3 + \dots}$,

and in general for the rth day it is $\dfrac{Z_r + Z_{r+1} + \dots}{\Sigma rZ_r}$

The frequencies of occurrence, where N is the total number of infected ships, and only a single case was taken on board, are—

$$F_1 = \frac{N}{\Sigma rZ_r}(Z_1 + Z_2 + Z_3 + Z_4 + \dots),$$

$$F_2 = \frac{N}{\Sigma rZ_r}(Z_2 + Z_3 + Z_4 + \dots),$$

$$F_3 = \frac{N}{\Sigma rZ_r}(Z_3 + Z_4 + \dots),$$

$$F_4 = \frac{N}{\Sigma rZ_r}(Z_4 + \dots\dots);$$

and these from the Australian statistics are equal to 64, 17, 5, and [illegible] respectively.

But the sum of probabilities of all possible incubations is certainty, *i.e.*, $Z_1 + Z_2 + Z_3 + \ldots\ldots = 1$, hence we may write

$$F_1 = \frac{N}{\Sigma r Z_r} = 64,$$

$$F_2 = \frac{N}{\Sigma r Z_r}(1 - Z_1) = 17,$$

$$F_3 = \frac{N}{\Sigma r Z_r}(1 - Z_1 - Z_2) = 5,$$

$$F_4 = \frac{N}{\Sigma r Z_r}(1 - Z_1 - Z_2 - Z_3) = 2.$$

By simple algebra we have

$$Z_1 = 0{\cdot}734,$$
$$Z_2 = 0{\cdot}186,$$
$$Z_3 = 0{\cdot}047,$$
$$Z_4 = 0{\cdot}031.$$

Also introducing these values into equations (1) we find the fundamental probabilities

$$p_1 = 0{\cdot}734,$$
$$p_2 = 0{\cdot}699,$$
$$p_3 = 0{\cdot}587,$$
$$p_4 = 0{\cdot}937.$$

As the last two values are relatively unreliable on account of the small integral values from which they are calculated, it seems reasonable to assume, as a first approximation, that p_r is constant, within the range of observation.

Equation (1) can now be reduced, and we have $Z_r = p\,(1-p)^{r-1}$. Also as $\Sigma r Z_r = p\{1 + 2(1-p) + 3(1-p)^2 + \ldots\}$

$$= p\left\{\frac{1}{p} + \frac{1-p}{p^2}\right\} = \frac{1}{p},$$

and $(Z_r + Z_{r+1} + \ldots\ldots\ldots) = p\,(1-p)^{r-1} + p(1-p)^r + p(1-p)^{r+1} + \ldots,$

$$= p(1-p)^{r-1}\left\{1 + (1-p) + (1-p)^2 + \ldots\right\}$$

$$= p(1-p)^{r-1}\frac{1}{p} = (1-p)^{r-1}.$$

the general form of equations (2) becomes—

$$F_r = Np(1-p)^{r-1} = NZ_r \quad \ldots\ldots\ldots\ldots\ldots(3)$$

The best value of p can now be obtained by moments,

$$\text{for } \Sigma rF_r = N \Sigma rZ_r = \frac{N}{p}$$

$$\therefore p = \frac{N}{\Sigma rF_r}$$

The calculation is as follows :—

	F_r	rF_r
$r = 1$	64	64
$= 2$	17	34
$= 3$	5	15
$= 4$	2	8
$N =$	88	$\Sigma rF_r = 121$

hence $p = \frac{88}{121} = 0{\cdot}727$.

Consequently by (3)—

	Observed.	Calculated to nearest integer.
$F_1 = 63{\cdot}98$	64	64
$F_2 = 17{\cdot}47$	17	17
$F_3 = 4{\cdot}77$	5	5
$F_4 = 1{\cdot}30$	2 } 2	2
$F_5 = 0{\cdot}36$	0 }	

The values of Z_r are

$Z_1 = 0{\cdot}727$,
$Z_2 = 0{\cdot}189$,
$Z_3 = 0{\cdot}054$,
$Z_4 = 0{\cdot}014$.

The mean incubation period from these figures is 32·71 hours with a probable error of about one hour.

The validity of the assumption that only one case was introduced into each ship may now be tested. If more than one case were introduced the resulting epidemic would probably be larger than if only one were taken on board. Consequently if the statistics were divided into classes according to the total number of cases which occurred, the non-validity of our assumption would be expected to declare itself in perturbations amongst the higher classes. The following figures show that there is little evidence of this, and that consequently the assumption may be accepted. This is the only way in which

differences in size, amongst the populations of the ships can affect the argument.

		Calculated.	Observed.	Calc. to nearest integer.	
Ships with one case only	F_1	20·47	21	20	
	F_2	6·30	5	6	p = 0·682
	F_3	1·94	3	2	
	F_4	0·60	1	1	
,, ,, 2—5 cases	F_1	22·14	21	22	
	F_2	3·99	5	4	p = 0·818
	F_3	0·72	1	1	
	F_4	0·13	0	0	
,, ,, 6—10 cases	F_1	8·65	9	9	
	F_2	1·85	1	2	p = 0·786
	F_3	0·40	1	0	
,, more than 10 cases	F_1	8·0	8	8	
	F_2	2·67	3	3	
	F_3	0·8	0	1	p = 0·67
	F_4	0·3	1	0	

In the above it has been assumed that variations in the chance of an individual acquiring infection were negligible during the four days prior to leaving port.

SUMMARY.

(1) From the times of occurrence of first cases of influenza, after the departure from port of the ships under consideration, it appears that the probability of a patient showing symptoms (irrespective of whether he showed them previously or not) does not significantly vary during the first four days of his infection.

(2) If this probability be taken as constant during these days, then the following results are obtained:—

(*a*) Ships in which the first case appeared

	Observed.	Calculated to nearest integer.
On the 1st day after leaving port	64	64
,, ,, 2nd ,, ,, ,, ,,	17	17
,, ,, 3rd ,, ,, ,, ,,	5	5
more than 3 days ,, ,,	2	2

(*b*) It also follows that of 100 cases of influenza, it is to be expected that 72·7 would have incubation periods of 1 day

18·9	,,	,,	,,	,,	,,	2	days
5·4	,,	,,	,,	,,	,,	3	,,
1·4	,,	,,	,,	,,	,,	4	,,

the mean incubation period being 32·7 hours.

(3) It the probability of a case being infective be related to the probability of symptoms being apparent, then the initial period of non-infectivity in influenza must be of short duration, which is a point of considerable epidemiological significance.
